# Disseminated Medulloblastoma in a Child with Germline *BRCA2* 6174delT Mutation and without Fanconi Anemia

**DOI:** 10.3389/fonc.2015.00191

**Published:** 2015-08-27

**Authors:** Jingying Xu, Ashley Sloane Margol, Anju Shukla, Xiuhai Ren, Jonathan L. Finlay, Mark D. Krieger, Floyd H. Gilles, Fergus J. Couch, Meraj Aziz, Eric T. Fung, Shahab Asgharzadeh, Michael T. Barrett, Anat Erdreich-Epstein

**Affiliations:** ^1^Division of Hematology-Oncology, Department of Pediatrics, Children’s Hospital Los Angeles, Los Angeles, CA, USA; ^2^Department of Pediatrics, Keck School of Medicine, University of Southern California, Los Angeles, CA, USA; ^3^Affymetrix, Santa Clara, CA, USA; ^4^Division of Neurosurgery, Children’s Hospital Los Angeles, Los Angeles, CA, USA; ^5^Department of Neurological Surgery, Keck School of Medicine, University of Southern California, Los Angeles, CA, USA; ^6^Department of Pathology, Children’s Hospital Los Angeles and Keck School of Medicine, University of Southern California, Los Angeles, CA, USA; ^7^Department of Laboratory Medicine and Pathology, Mayo Clinic, Rochester, MN, USA; ^8^Translational Genomics Research Institute (TGen), Phoenix, AZ, USA

**Keywords:** *BRCA2* 6174delT, Fanconi anemia, group 4 medulloblastoma, *MYC*, medulloblastoma cell lines

## Abstract

Medulloblastoma, the most common malignant brain tumor in children, occurs with increased frequency in individuals with Fanconi anemia who have biallelic germline mutations in *BRCA2*. We describe an 8-year-old child who had disseminated anaplastic medulloblastoma and a deleterious heterozygous *BRCA2* 6174delT germline mutation. Molecular profiling was consistent with Group 4 medulloblastoma. The posterior fossa mass was resected and the patient received intensive chemotherapy and craniospinal irradiation. Despite this, the patient succumbed to a second recurrence of his medulloblastoma, which presented 8 months after diagnosis as malignant pleural and peritoneal effusions. Continuous medulloblastoma cell lines were isolated from the original tumor (CHLA-01-MED) and the malignant pleural effusion (CHLA-01R-MED). Here, we provide their analyses, including *in vitro* and *in vivo* growth, drug sensitivity, comparative genomic hybridization, and next generation sequencing analysis. In addition to the *BRCA2* 6174delT, the medulloblastoma cells had amplification of *MYC*, deletion at Xp11.2, and isochromosome 17, but no structural variations or overexpression of GFI1 or GFI1B. To our knowledge, this is the first pair of diagnosis/recurrence medulloblastoma cell lines, the only medulloblastoma cell lines with *BRCA2* 6174delT described to date, and the first reported case of a child with medulloblastoma associated with a germline *BRCA2* 6174delT who did not also have Fanconi anemia.

## Introduction

Medulloblastomas are the most common malignant brain tumors in children. While advances in diagnosis and therapy have improved long-term survival of children with standard-risk medulloblastoma, children with high-risk disease continue to fare poorly (1, 2). Grouping according to histology shows that patients with desmoplastic/nodular morphology have a better prognosis than those with classic morphology, and both are better than medulloblastomas with large cell/anaplastic histology (3). More recent classifications, which divide medulloblastomas into four molecular subgroups [WNT, Sonic Hedgehog (SHH), Group 3 and Group 4] show that tumors with genomic alterations in SHH and WNT signaling pathways overall had better prognosis than Groups 3 and 4 medulloblastomas (4–7).

*BRCA2* (breast cancer 2, early onset), identified in 1994, is a tumor suppressor that functions in homologous recombination and double-stranded DNA repair (8, 9). Deleterious germline mutations in *BRCA2* may confer up to 84% risk of breast cancer and 27% risk of ovarian cancer in women, and are frequently associated with Ashkenazi Jewish decent (*Am. J. Hum. Genetics* 62:676–689, 1998) (10). The carrier rate of the *BRCA2* 6174delT founder mutation in individuals of Ashkenazi Jewish decent is estimated to be 0.9% (CI 0.6–1.5%), and is calculated to have arisen approximately 90 generations ago (11, 12). Over a decade ago, Howlett et al. reported that *BRCA*2 was the long sought-after gene mutated in the germline of patients with Fanconi anemia complementation group D1 (FANCD1) (13). Fanconi anemia is an autosomal recessive cancer susceptibility disorder caused by mutation in 1 of 13 known genes that function in genomic maintenance (13, 14). Children with Fanconi anemia who develop solid tumors, including medulloblastomas, frequently have biallelic germline mutations in *BRCA2* (15–18). To date, however, there has been no report of a medulloblastoma associated with a deleterious *BRCA2* germline mutation in a patient without Fanconi anemia.

Here, we report for the first time on a child carrying a familial heterozygous *BRCA2* 6174delT germline mutation, who presented with metastatic medulloblastoma. We also provide characterization of his tumor and the unique pair of medulloblastoma cell lines generated from it at diagnosis and at the time of systemic metastatic recurrence.

## Materials and Methods

### Cell culture

Primary tumor was obtained at initial surgery before any chemotherapy or irradiation. Recurrent tumor cells were obtained from a malignant pleural effusion that was removed for clinical indication at the time of second recurrence. Primary tumor tissue was minced and cultured in Dulbecco’s Modified Eagle Medium: Nutrient Mixture F-12 (DMEM/F-12) medium with B-27 supplement (Invitrogen, CA, USA), EGF (20 ng/ml, Invitrogen, CA, USA), and bFGF (20 ng/ml, Cell Sciences, MA, USA) in a standard humidified incubator at 37°C in 5% CO_2_/95% atmospheric air. Gentamicin (50 μg/ml) was used for the initial 2 weeks of culture and then withdrawn to facilitate detection of mycoplasma. Tumor cells from the malignant pleural effusion were cultured similarly except without mincing. Small tandem repeat (STR) profile (ATCC) authenticated that both cell lines originated in the same individual. Both the original medulloblastoma cell line (CHLA-01-MED) and its subsequent pleural fluid recurrence cell line (CHLA-01R-MED) are now available through the ATCC repository (CRL–3021 and CRL-3034, respectively). Lysates from other cell lines were UW-228-2, D283MED medulloblastomas, CHLA-02-ATRT (ATCC CRL-3020) (19, 20), LN229 GBM, and 293T cells.

### Chromosome analysis

*BRCA2* mutation analysis was by PCR and direct DNA sequencing. Copy number analysis was performed on second passage cells from the tumor at diagnosis (CHLA-01-MED) and in recurrent tumor cells obtained directly from the malignant pleural effusion. DNAs were extracted using Qiagen micro kits (Qiagen Valencia, CA, USA). Concentrations and quality were determined by fluorometry (QuBit^®^, Life Technologies). Subsequently 1 μg of genomic DNA from each sample and a 1 μg aliquot of commercial 46,XX reference template (Promega Madison, WI, USA) were digested with DNase I then labeled with Cy-5 dUTP and Cy-3 dUTP respectively, using a BioPrime labeling kit (Invitrogen, Carlsbad, CA, USA). All labeling reactions were assessed using a Nanodrop assay (Nanodrop, Wilmington, DE, USA) prior to mixing and hybridization to comparative genomic hybridization (CGH) arrays with 400,000 oligonucleotide features (Agilent Technologies, Santa Clara, CA, USA). Microarray slides were scanned using an Agilent 2565C DNA microarray scanner and the images were analyzed with Agilent Feature Extraction software version 10.7 (FE 10.7) using default settings according to the supplier’s recommendations. Log_2_ ratios of fluorescent signals and corresponding log_2_ ratio errors were calculated from the log_10_ output of FE 10.7 for each hybridization and analyzed in GenomeWorkbench 7.0.

Genome-wide copy number determination for the diagnostic formalin-fixed paraffin embedded (FFPE) sample was carried out using OncoScan system utilizing molecular inversion probe (MIP) technology (Affymetrix, Santa Clara, CA, USA). In brief, three scrolls of FFPE material (20 μm thickness) were sent to Affymetrix for DNA extraction using the OncoScan FFPE assay kit and for generation of MIP probes followed by hybridization on the Affymetrix MIP 330K platform. The data were analyzed using APT tools and imported into OncoScan Nexus Express Software (Biodiscovery, El Segundo CA, USA) for visualization and analysis.

### Cytotoxicity assays

Cells were seeded in 150 μl Iscove’s Modified Dulbecco’s Medium (IMDM) supplemented with 20% fetal bovine serum, 4 mM l-glutamine, and 0.1% ITS stock solution (ITS is insulin, selenium, and transferrin, Mediatech, VA, USA) at 12,000 cells/well in 96-well plates to facilitate attachment and spreading. After 6 h drugs were added at doses that are within clinically achievable plasma concentrations (21–23) and cells were incubated an additional 96 h. To measure cytotoxicity, fluorescein diacetate (FDA) and eosin Y (to quench background fluorescence) were added to cells at final concentration of 10 μg/ml, and incubated 30 min in the dark. Total fluorescence per well was measured using the DIMSCAN assay system, after digital thresholding, to further eliminate background fluorescence (24, 25). Results are expressed as survival fraction compared to control cells. The concentration of drug that was cytotoxic and/or growth inhibitory for 90% of cells (IC_90_) was calculated using the software “Calcusyn” (Biosoft, Cambridge, UK).

### Informed consent

Following informed consent, the patient was enrolled on the Children’s Hospital Los Angeles Neural Tumors Registry, as approved by the institutional Committee for Clinical Investigations.

### *In vivo* tumor studies

Mouse studies were performed according to a protocol approved by the Children’s Hospital Los Angeles Institutional Animal Care and Use Committee. Intracranial implantation was done using a stereotaxic frame as previously described (26). Briefly, 2 × 10^5^ cells in 2 μl DMEM/F-12 were injected into the right caudate/putamen to a depth of 3.3 mm in 4- to 6-week-old female NOD/SCID mice (Bar Harbor, ME, USA). Mice were monitored daily and intracranial tumor size was followed by magnetic resonance imaging (MRI) at 1–3 weeks intervals. Mice were euthanized when the tumor reached a diameter of 4–5 mm or when the mice displayed signs of distress, whichever occurred earlier. Brains of the mice were harvested following vascular perfusion (PBS followed by buffered 4% paraformaldehyde) under deep anesthesia. Half the brain, cut through the tumor, was snap frozen in OCT and the other half fixed in 4% buffered paraformaldehyde for paraffin embedding. Sections (6 μm) were cut and stained with hematoxylin and eosin (H&E).

Magnetic resonance imaging in mice was performed as described (26) using a 7-T Bruker Pharmascan scanner (Bruker, Ettlingen, Germany) with a 19 mm radiofrequency coil (27). About 15–20 min prior to scanning, mice were injected intraperitoneally with 100 μl gadopentatate dimeglumine (Magnevist, Bayer HealthCare Pharmaceuticals, Wayne, NJ, USA). Mice were then sedated with 5% isoflurane and held in an anesthetic plane with 1.5–2% for the entirety of the scans. Largest tumor axial dimensions were measured.

### Reagents

Cisplatin (CDDP, APP Pharmaceutical LLC, IL, USA), etoposide (ETOP, Bedford Laboratories, OH, USA), methotrexate (MTX, Hospira, IL, USA), and Vincritine (VCR, APP Pharmaceutical LLC, IL, USA) were obtained through the Children’s Hospital Los Angeles pharmacy. PARP inhibitor AZD2281 was from Selleck (Houston, TX, USA). FDA was from Eastman Kodak Company (Rochester, NY, USA). Eosin Y was from Sigma Chemical Co. (St. Louis, MO, USA). All other reagents were from Sigma unless specified otherwise.

### RNA preparation and next generation sequencing and analysis

RNA was extracted from each sample of interest with the RNeasy minikit (Qiagen) according to established protocols. We used the NuGen Ovation RNASeq System v2 to generate double-stranded cDNA from 10–50 ng aliquots of RNA. These were subsequently amplified using NuGen’s SPIA linear amplification process. Amplified products were cleaned using Qiagen’s QIAquick PCR Purification Kit and quantitated using Invitrogen’s Quant-iT Picogreen. One microgram of amplified cDNA was fragmented on the Covaris E210 to a target size of 300 bp. Illumina’s TruSeq DNA Sample Preparation Kit was used to prepare libraries from 1 μg amplified cDNA according to the manufacturer’s protocol. Final libraries were quantitated by Qubit (Invitrogen) and evaluated on the Agilent Bioanalyzer. Libraries were clustered onto paired end flow cells at concentrations of 12–16 pM to generate total densities of 750,000–850,000/mm^2^ using the Illumina cBot and HiSeq Paired end cluster kit version 3. The flow cells were sequenced as 83 × 2 paired end reads on the Illumina HiSeq using the TruSeq SBS sequencing kit version 3 and HiSeq control software version 2.0.10 software. Bcl conversion was performed using Illumina’s Bclconverter software. All sequencing data were checked for quality using cycle-by-cycle quality plots. RNAseq data were aligned to human genome assembly GRCh37 (hg19, NCBI build 37) using the STAR aligner (28). HTSeq was then used to further process the output from STAR aligner and format the data for DESeq, which was used to identify differentially expressed genes (29, 30). These genes were then mapped onto pathways using GeneGo. All array comparative genomic hybridization (aCGH) and expression data in this paper have been deposited at the National Center for Biotechnology Information Gene Expression Omnibus; accession number GSE70003.

### Western blot analysis

Cells were collected using cell scraper, re-suspended in 1× sodium dodecyl sulfate (SDS) sample buffer, sonicated, boiled for 5 min, and resolved by SDS–polyacrylamide gel electrophoresis (SDS-PAGE) gel. Primary antibodies for western blots were anti-BRCA2, rabbit polyclonal (1:1,000; Cat# A303-434A-T, Bethyl Laboratories, TX, USA); anti-p21, goat polyclonal (1:500; Santa Cruz, CA, USA); anti-p53, mouse monoclonal (1:1000; BD Biosciences, CA, USA); anti-GAPDH, mouse monoclonal (1:20,000; Santa Cruz Biotechnology, CA, USA). Detection was by ECL (Amersham Biosciences, Piscataway, NJ, USA) and densitometry was by ImageJ software (National Institutes of Health).

### Statistical analysis

Cell culture experiments were performed using at least three replicates, except that DIMSCAN cytotoxicity assays used 12 replicates. Experiments were repeated three times unless indicated otherwise. *In vitro* cytotoxicity data were analyzed and graphed using the DIMSCAN Data Analyzer Program and Prism GraphPad 4.0c. Results with a *p*-value <0.05 were considered significant.

## Results

### Medulloblastoma in child with familial heterozygous germline *BRCA2* 6174delT mutation and without fanconi anemia

**Figure 1:**
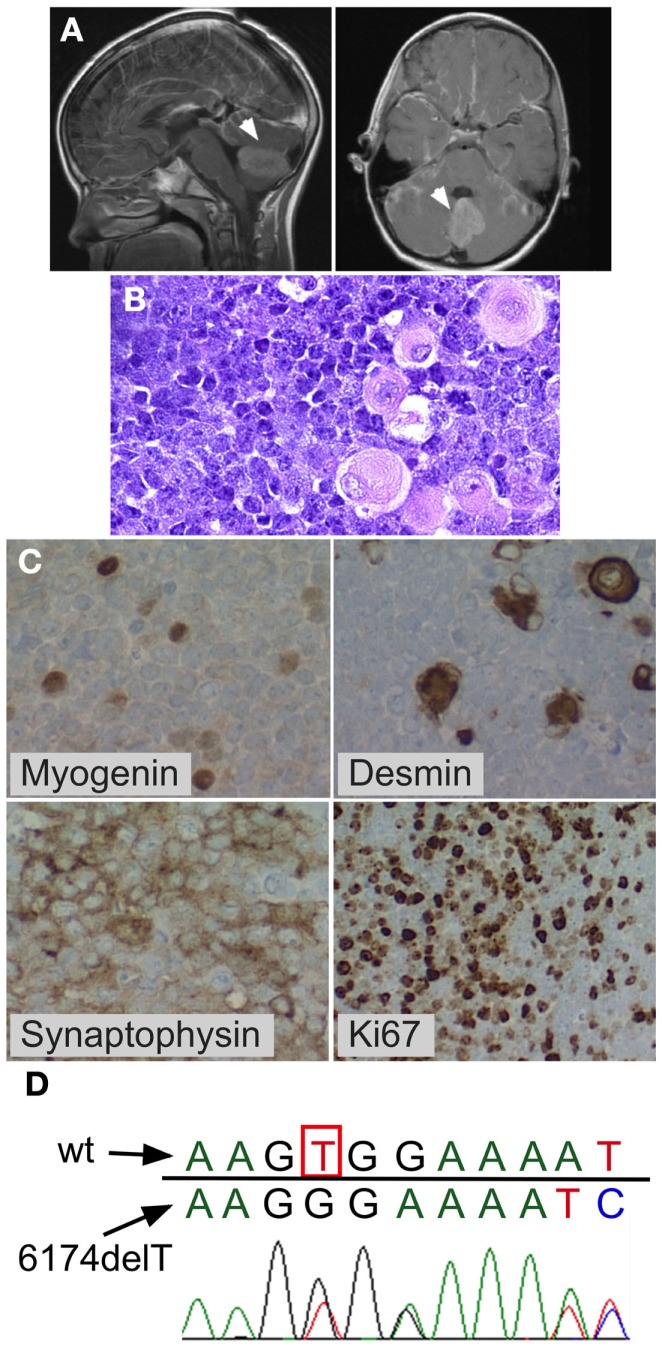
**Medulloblastoma with myogenic features**. Posterior fossa anaplastic medulloblastoma with myogenic features. **(A)** MRI at diagnosis showing posterior fossa mass. **(B)** H&E stain showing anaplastic medulloblastoma. Note the large cells with pink cytoplasm. **(C)** *BRCA2* sequencing of peripheral blood mononuclear cells from the patient showed the wildtype nucleotide 6174T (c.5946T) on one allele (red peak), which is lost in the second allele, resulting in a G nucleotide (black peak) at the same position (arrow) and a downstream frameshift. **(D)** Immunohistochemistry demonstrating myogenic differentiation (cells positively staining for myogenin and desmin), as well as positive synaptophysin, and high mitotic index (Ki67).

An 8–year-old previously healthy male of 97th percentile height, 90th percentile weight and normal anatomy presented following a 2-week history of worsening blurry vision, dizziness, and emesis, culminating in increasing somnolence. A brain MRI showed cerebellar mass with diffuse leptomeningeal enhancement consistent with leptomeningeal metastases. The spine MRI showed disseminated leptomeningeal disease of the entire cord including the cauda equina region. The posterior fossa mass was completely resected. Due to persistent high cerebrospinal fluid (CSF) output from the post-operative external ventricular drain in subsequent days, a ventriculoperitoneal shunt was placed. Lumbar CSF cytology showed 3,000 tumor cells per microliter. Pathological evaluation showed anaplastic medulloblastoma with myogenic differentiation (Figure 1). Molecular subgrouping was consistent with Group 4 medulloblastomas using a medulloblastoma specific 31-gene TaqMan Low Density Array (TLDA) classifier (31). Chemotherapy was initiated according to the “Head Start” III protocol with vincristine, cisplatin, cyclophosphamide, etoposide, and high-dose methotrexate (32, 33). However, after two cycles, a new tumor nodule was apparent on a spine MRI and therapy was changed to craniospinal irradiation (36 Gy craniospinal, 18 Gy IMRT posterior fossa boost, and 9 Gy spinal cord boost) combined with concomitant daily carboplatin, with resultant shrinkage of the nodule. The child tolerated both the intensive chemotherapy and the craniospinal irradiation without unexpected or inordinate toxicities. Three months after the end of irradiation (8 months after diagnosis), the patient developed symptomatic malignant pleural and peritoneal effusions that reaccumulated repeatedly after drainage and led to his demise several weeks after their development.

The child’s family history was significant for maternal bilateral breast cancer developing at the age of 38 years, breast cancer in one of her two sisters, and colon cancer in their father. *BRCA* testing had previously demonstrated a deleterious heterozygous germline *BRCA2* 6174delT frameshift mutation in the mother and in her sister who had breast cancer. Family was non-Jewish Caucasians of European decent and they were not aware of Ashkenazi Jewish ancestors in recent generations. The patient’s peripheral blood mononuclear cells showed one wildtype *BRCA2* allele and *BRCA2* 6174delT on the other allele (Figure 1D). The family history, the patient’s prior normal medical history, his pre-tumor normal blood counts and physical exam, his age at medulloblastoma diagnosis (8 years), normal tolerance to intensive chemotherapy, and normal diepoxybutane (DEB) chromosome breakage study did not support the presence of Fanconi anemia in this child.

### New paired medulloblastoma cell lines: CHLA-01-MED and CHLA-01R-MED

**Figure 2:**
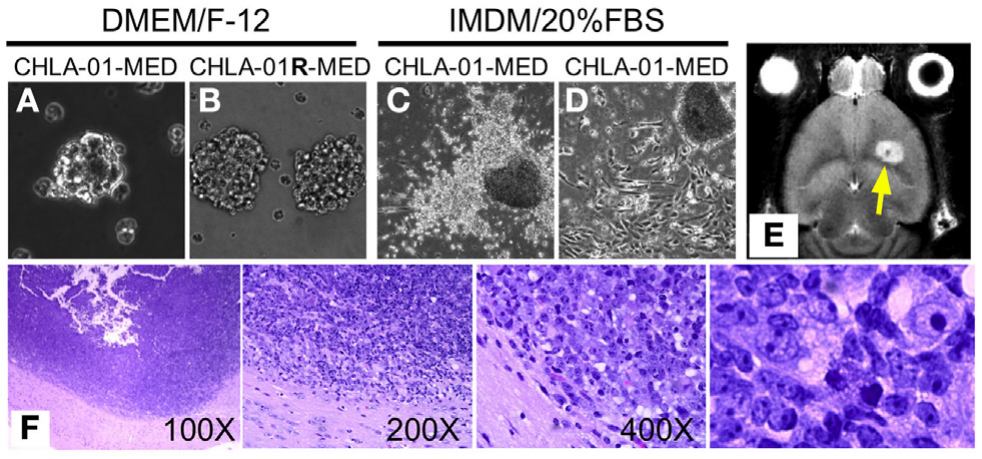
**CHLA-01-MED and CHLA-01R-MED medulloblastoma cell lines grow as neurospheres and form intracranial medulloblastoma *in vivo***. Cell lines shown were generated from the original tumor (CHLA-01-MED) and the pleural effusion recurrence (CHLA-01R-MED). **(A,B)** The two cell lines grow as neurospheres when cultured under non-adherent serum-free conditions; **(C,D)** when seeded in FBS-containing medium, low-passage CHLA-01-MED attach and spread. (CHLA-01R-MED do not; not shown); **(E,F)** low-passage CHLA-01-MED injected into the frontal lobe of NOD/SCID mice developed tumors. **(E)** MRI of mouse brain with CHLA-01-MED tumor; **(F)** H&E of a CHLA-01-MED tumor. Right panel is inset from the 400× panel.

Two cell lines were established from the medulloblastoma of this patient: CHLA-01-MED from the primary tumor resected at diagnosis, and CHLA-01R-MED from tumor cells in the malignant pleural effusion 8 months later, after he was treated with chemotherapy (vincristine, cisplatin, cytoxan, etoposide, and methotrexate) followed by craniospinal irradiation with concomitant daily carboplatin. After securing low-passage cells, both cell lines, maintained in DMEM/F-12 medium supplemented with B-27 supplement, EGF (20 μg/ml) and bFGF (20 μg/ml), have been cultured continuously for more than 2 years and can thus be classified as permanent cell lines. Under these culture conditions, both CHLA-01-MED (Figure 2A) and CHLA-01R-MED (Figure 2B) were non-adherent, formed loose spheres that were easily dispersed, and maintained 30–50% viability. Low-passage CHLA-01-MED (less than passage 10), when grown in IMDM with 20% FBS, attached to the tissue culture surface within 1 day. Single cells exited the sphere within a few days and grew as monolayer that rapidly proliferated (Figure 2C). However, after approximately 3 months, cells in these adherent FBS-containing cultures ceased to proliferate. With increasing passages, CHLA-01-MED required longer to attach and form a monolayer (Figure 2D) until they were no longer able to do so, even in FBS-containing culture conditions. The cell line from the malignant pleural effusion (CHLA-01R-MED) continuously grew in suspension even after months in IMDM/20% FBS, and was unable to grow as monolayer (data not shown). Low passages of both matched cell lines have been deposited in the ATCC repository (Manassas, VA, USA).

### CHLA-01-MED forms tumors *In vivo* in mice

Utility of tumor cell lines as pre-clinical models may be enhanced if they can grow into tumors *in vivo* in mice. CHLA-01-MED cells (first passage of the cells from the resected tumor) injected intracranially into brains of NOD/SCID mice generated medulloblastomas, which were detected by MRI in four of the five mice tested starting 44 days after injection (Figure 2E, arrowhead). The morphology of these mouse tumors recapitulated the histology of the patient’s tumor (Figure 2F), both were composed of densely packed primitive cells with round-to-oval hyperchromatic nuclei surrounded by scant cytoplasm and consistent with medulloblastoma.

### Chromosomal and array comparative genomic hybridization analysis of the tumor and its cell lines

Karyotype analysis of CHLA-01-MED cells (cell line from original tumor at diagnosis) showed 42–46 chromosomes and two consistent chromosomal rearrangements: der(17)t(1;17)(q11;q25) and der(18)(17;18)(q11.2;p11.2). Abnormalities of chromosome 17 are common in both Group 3 and Group 4 medulloblastomas (6, 34–36). The CHLA-01R-MED cell line, generated from the malignant pleural effusion at recurrence, showed diploid cells with a del(11)(q13.3) in all examined cells. The 11q breakpoint in the recurrence cell line was also present in malignant cells analyzed directly from the pleural effusion, supporting its origin in the recurrent tumor rather than a product of cell culture. STR profile by ATCC (available on the ATCC website) confirmed that both lines originated in the same individual.

**Figure 3:**
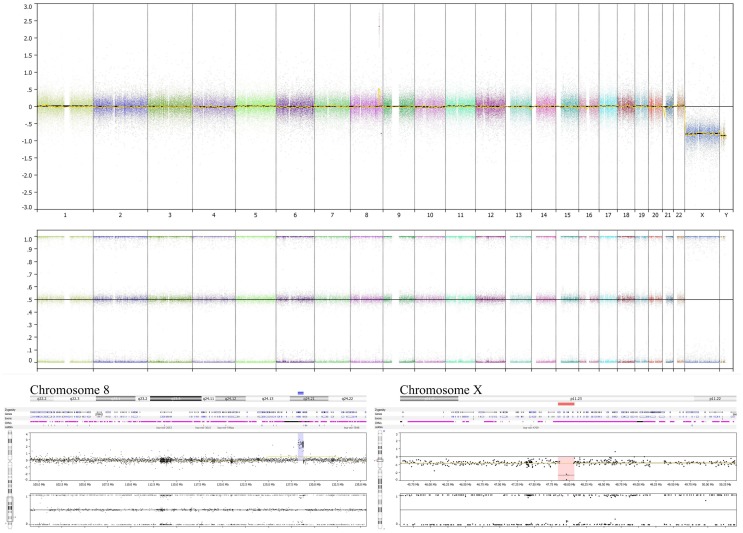
**Tumor specimen obtained at diagnosis has *MYC* amplification and deletion at Xp11.2**. OncoScan results using MIP technology to detect copy number and loss of heterozygosity information of the original diagnostic tumor FFPE material demonstrates the amplified *MYC* locus at 8q24.21 (lower left panel) along with deletion of the SSX6 region at Xp11.2 (lower right panel). The deletion at Xp11.2 in this boy’s FFPE tumor sample, without detection of a non-deleted signal, suggests that the FFPE specimen analyzed comprised nearly 100% tumor cells, without significant contribution from non-tumor cells. This analysis does not show chromosome 17 or 11q alterations (upper panel).

Genome copy number assessment of the FFPE tissue of this boy’s original tumor showed two significant events: amplification of the *MYC* locus and deletion at Xp11.2 (Figure 3).

**Figure 4:**
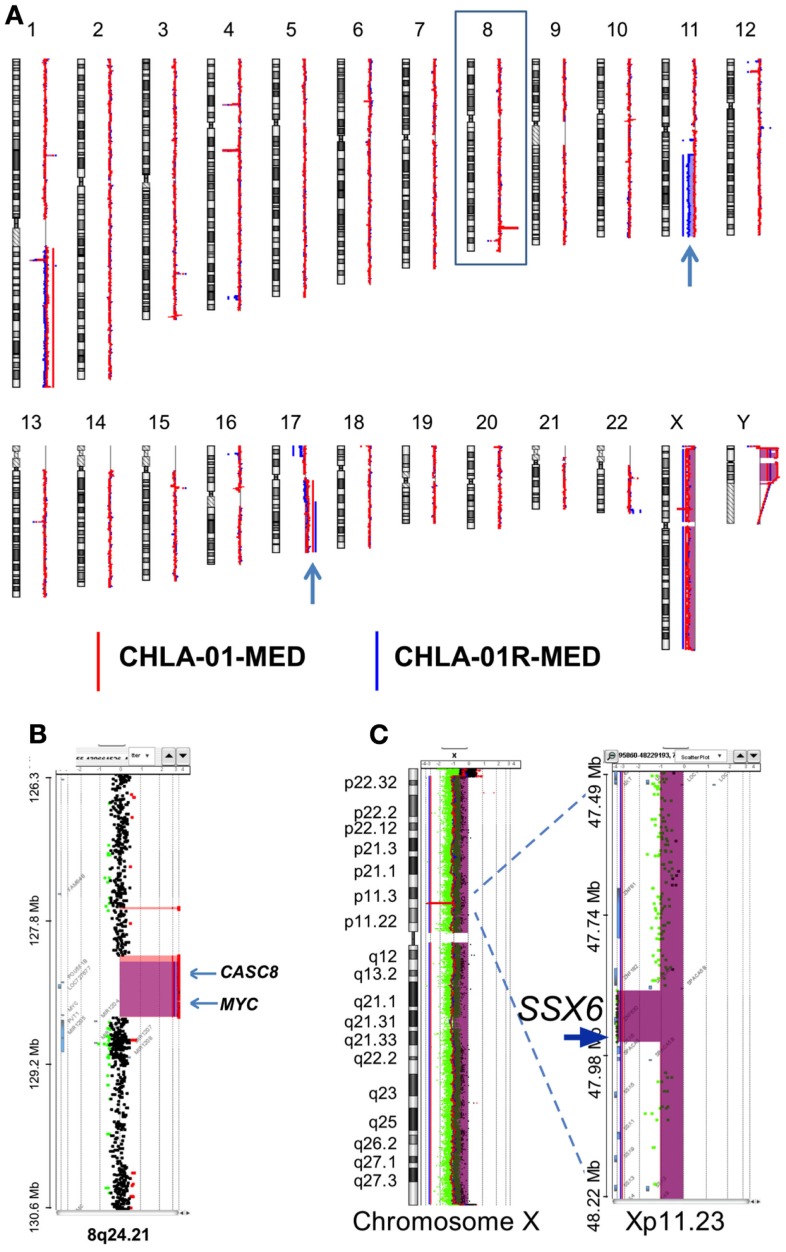
**CHLA-01-MED and CHLA-01R-MED cell lines have *MYC* amplification and deletion at Xp11.2**. Comparative genomic hybridization profiles of the cell lines generated from the original tumor (CHLA-01-MED) and the pleural effusion recurrence (CHLA-01R-MED). **(A)** Whole genome plots of CHLA-01-MED and CHLA-01R-MED. **(B)** High-level focal amplicon on chromosome 8q24.21 targets the *MYC* locus. Shaded areas denote copy number aberrations defined by ADM2 step gram algorithm (37). **(C)** CHLA-01-MED and CHLA-01R-MED show focal (88.8 kb) homozygous deletion at Xp11.2 targeting the Synovial Sarcoma X breakpoint gene *SSX6* (aCGH). Shaded areas and horizontal lines denote ADM2-defined copy number aberrant intervals. Results for both cell lines are displayed in each panel and highlight overlap of deleted region.

**Figure 5:**
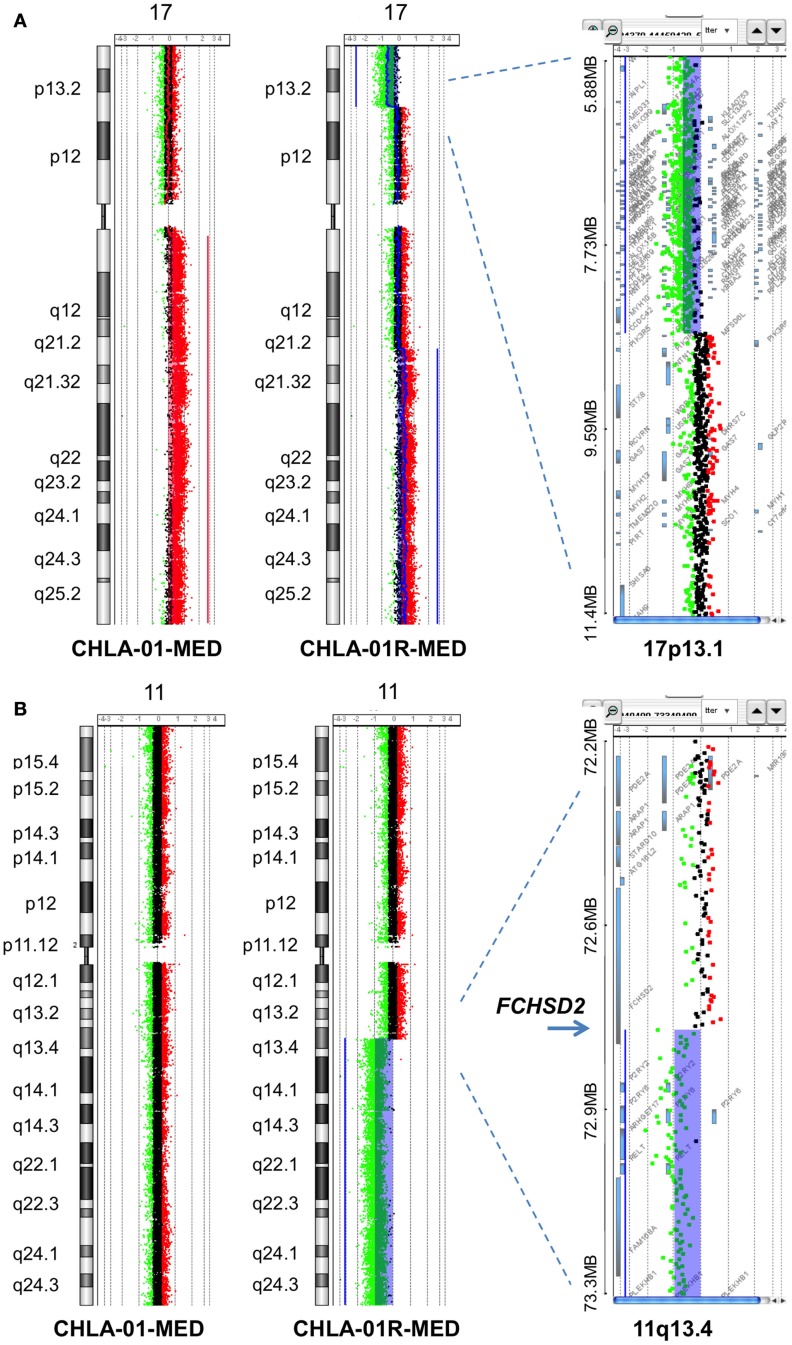
**Additional aberrations in CHLA-01-MED and CHLA-01R-MED: (A) distinct chromosome 17 aberrations in CHLA-01-MED and CHLA-01R-MED: CGH results show a unique deletion of 17p13.2-ptel and a gain of 17q21.2-qtel in CHLA-01R-MED (right and middle panels)**. In comparison, CHLA-01-MED had a gain of the entire q arm of chromosome 17 (left panel). Shaded areas and horizontal lines in each panel denote ADM2-defined copy number aberrant intervals. **(B)** CHLA-01R-MED, but not CHLA-01-MED, shows deletion of 11q13.4-qtel with breakpoint at chromosome 11q13.4 targeting *FCHSD2* (FCH and double SH3 domains protein 2) (aCGH). Shaded areas and horizontal lines in right and middle panels denote ADM2-defined copy number aberrant intervals. In comparison, there were no copy number aberrations detected on chromosome 11 in CHLA-01-MED (left panel).

Comparative genomic hybridization analysis of CHLA-01-MED (passage 2 cells from the original tumor), of cells from the malignant pleural effusion itself, and of the cell line generated from this pleural effusion (CHLA-01R-MED, passage 12), identified copy number aberrations consistent with the FFPE findings and the karyotype of each (Figure 4). The most significant aberrant copy number interval was a focal (536 Mb) high-level (log_2_ ratio >8) amplification of *MYC* in both CHLA-01-MED and CHLA-01R-MED (Figure 4B). Both diagnosis and recurrence cell lines from this boy’s medulloblastoma also showed the focal (88.8 kb) deletion at Xp11.2, which targeted the Synovial Sarcoma X breakpoint gene, *SSX6* (Figure 4). Other significant alterations were duplication of the q arms of chromosomes 1 and 17 in CHLA-01-MED and deletion of 11q13.4-qtel with breakpoint at chromosome 11q13.4 targeting *FCHSD2* (FCH and double SH3 domains protein 2) in CHLA-01R-MED (Figures 4A and 5). RNASeq analysis confirmed that *MYC* was highly expressed in both cell lines. Interestingly, the long non-coding RNA Cancer Susceptibility Candidate 8 (*CASC8*), which is included within the focal MYC amplicon of both original and recurrent cell lines, was one of the most significant and differentially expressed transcripts with an approximately 700-fold overexpression in CHLA-01R-MED compared to the primary CHLA-01-MED cell line.

Chromosome 17q gain was not detected in the analysis of the FFPE diagnostic tumor material, although it was present in passage 2 cells in culture from this original tumor (CHLA-01-MED cells) and in the pleural fluid cells obtained at the time of recurrence. This suggests that cells with 17q gain may have been a minority clone at diagnosis, which became rapidly prominent in culture and in the recurrence, or this difference may be due to differences in technique of analysis. The deletion in 11q, which was detected in the recurrence pleural effusion cells and their CHLA-01R-MED cell line but not in the cell line from the original tumor (CHLA-01-MED), was also not detected in the FFPE tissue from the original tumor, supporting that it originated in the recurrent tumor.

Northcott et al. recently showed that overexpression of GFI1 or GFI1B, associated with genomic lesions (duplication, breakpoints) around 1p22 and 9q34, respectively, act as oncogenes cooperating with *MYC* in Group 3 and Group 4 medulloblastomas (38). CHLA-01-MED and CHLA-01R-MED showed no obvious copy number aberrations involving either gene. The RNAseq results also indicate that GFI1 was not highly expressed and GFI1B transcripts were not present at all, indicating that GFI1 and GFI1B were not the *MYC-*cooperating genes in this medulloblastoma.

Last, analysis of *BRCA2* in CHLA-01-MED (second passage cells from the tumor at diagnosis) showed a *BRCA2* 6174delT frameshift mutation in one allele, similar to the *BRCA2* 6174delT germline mutation found in the germline of this patient and his mother. Western blotting of both cell lines showed a BRCA2-reactive protein band of similar size to other cell lines, albeit at amounts that seem lower (Figure S1 in Supplementary Material), possibly consistent with expression from one allele only. This supports that protein was made in the brain from the remaining allele of BRCA2. Interestingly, the UW-228-2 medulloblastoma cell line showed almost no anti-BRCA2 antibody reactivity, while the other medulloblastoma cell line, D283MED, had BRCA2 protein amounts comparable to the CHLA-02-ATRT, LN229 GBM, and 293T cell lines (Figure S1 in Supplementary Material).

### CHLA-01-MED and CHLA-01R-MED are resistant to commonly used chemotherapy

**Figure 6:**
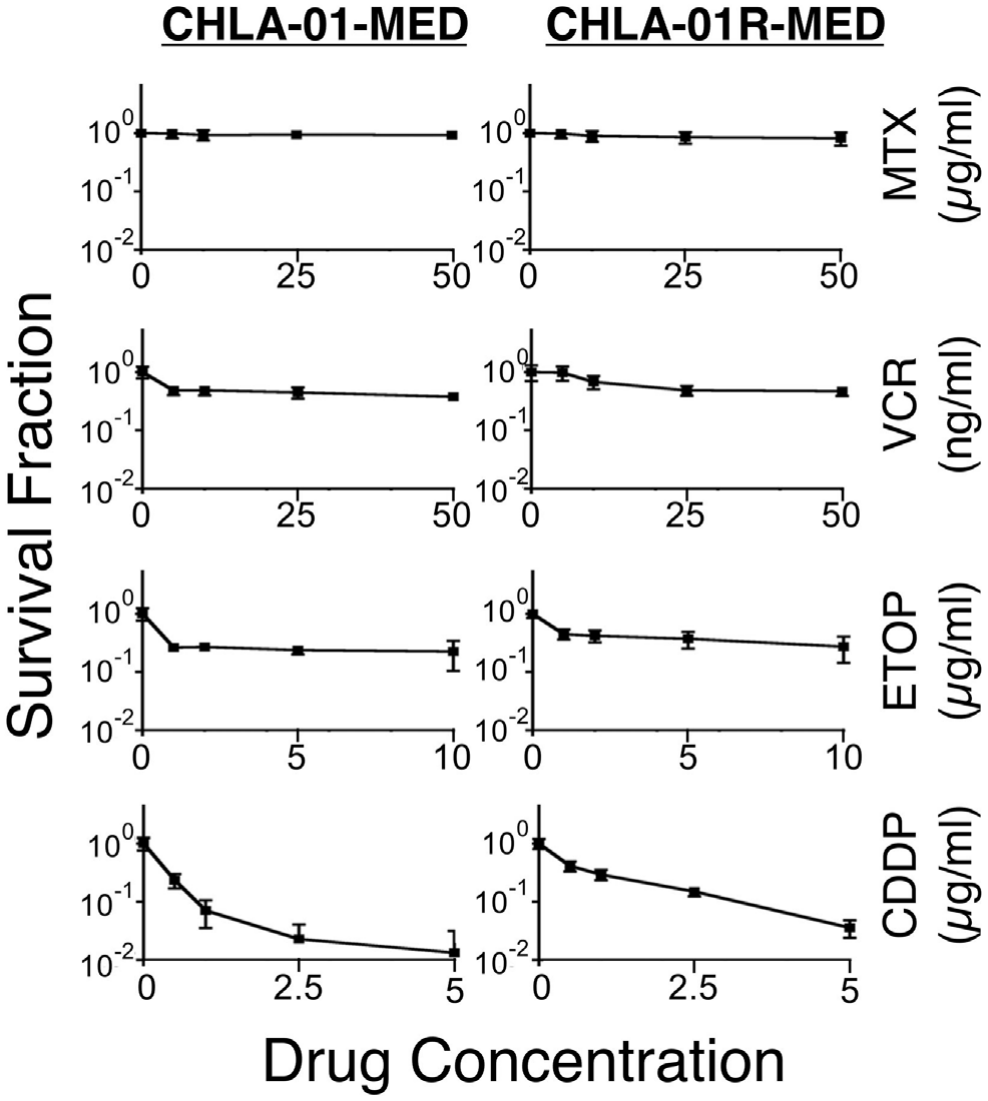
**CHLA-01-MED and CHLA-01R-MED cells are resistant to chemotherapy agents commonly used in medulloblastoma therapy**. Cells were incubated with drugs for 96 h and analyzed by DIMSCAN as described in the Section “Materials and Methods.” Each data point represents mean/SEM of 3 independent experiments, each performed in 12 replicates. CDDP, cisplatin; ETOP, etoposide; MTX, methotrexate; VCR, vincristine.

We next examined the cytotoxicity profile of the new cell lines using drugs commonly used in medulloblastoma therapy: etoposide, vincristine, methotrexate, and cisplatin (22, 39), all of which had been used in this patient. Cytotoxicity assays showed resistance of both CHLA-01-MED and CHLA-01R-MED to methotrexate, intermediate sensitivity to etoposide and vincristine, and better sensitivity to cisplatin (Figure 6; Table 1). The relative sensitivity to cisplatin may be associated with the BRCA2 mutation in these cells, which has been reported to increase sensitivity to this drug (40). The recurrence cell line, CHLA-01R-MED, had approximately twofold decreased sensitivity to cisplatin (Figure 6; Table 1), possibly reflecting additional mutations (41). Because this medulloblastoma had an oncogenic mutation in *BRCA2* (6174delT), we also tested its *in vitro* response to a PARP1 inhibitor, thought to induce synthetic lethality in *BRCA2*-mutated breast cancer (42). However, both cell lines were resistant to the PARP inhibitor AZD2281 (up to 1 μM) and to its combination with etoposide (data not shown). In summary, this new pair of medulloblastoma cell lines is sensitive to cisplatin but resistant to vincristine, etoposide, methotrexate, and the PARP inhibitor, AZD2281.

**Table 1:** **IC_90_ of both medulloblastoma cell lines to commonly used chemotherapy in medulloblastoma**.

IC_90_	Etoposide (μg/ml)	Vincristine (ng/ml)	Cisplatin (μg/ml)	Methotrexate (μg/ml)
CHLA-01-MED	>10	>50	0.89 ± 0.17	>50
CHLA-01R-MED	>10	>50	1.86 ± 0.67	>50

### CHLA-01-MED and CHLA-01R-MED have functional p53

**Figure 7:**
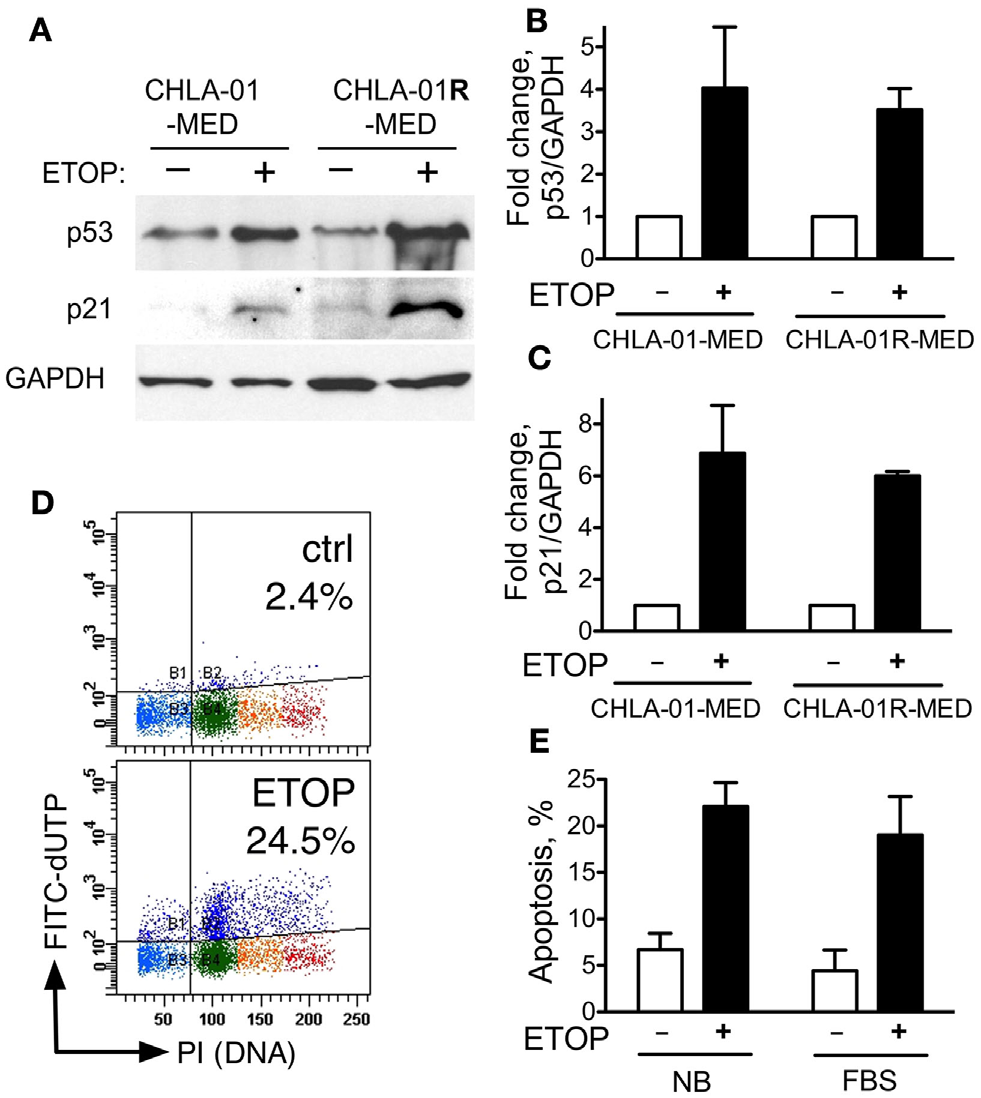
**CHLA-01-MED and CHLA-01R-MED cells have functional p53**. CHLA-01-MED and CHLA-01R-MED were incubated with 5 μg/ml etoposide for 24 h. Cells were analyzed by western blotting **(A)** and flow cytometry **(D,E)**. **(B,C)** are densitometric analyses of **(A)**. Results are mean/SEM from two independent experiments.

The activity of some chemotherapy drugs is linked to p53-dependent inhibition of proliferation or induction of apoptosis (21, 43). CGH analysis identified chromosome 17 aberrations in both of our new medulloblastoma cell lines. These included duplication of 17q and an additional distinct breakpoint in CHLA-01R-MED (Figures 4A and 5B). CHLA-01R-MED also had a unique deletion at 17p13.2-ptel that included the *TP53* locus. However, there were no mutations in the other copy of *TP53*. We examined the p53 functionality of these cell lines. At baseline, CHLA-01-MED and CHLA-01R-MED both expressed p53 protein (Figure 7A). Exposure to the DNA damaging agent etoposide (5 μg/ml) increased p53 protein expression in both cell lines as anticipated (Figures 7A,B). Etoposide also increased p21 protein, a downstream target of p53, indicating an intact sensing and response of the DNA damage-induced p53 pathway in both cell lines (Figure 7). Etoposide also induced apoptosis in CHLA-01-MED cells cultured in non-adherent (DMEM/F-12 + B-27 + EGF + bFGF) as well as in adherent (IMDM + FBS) conditions (Figures 7D,E; measured as increased FITC-dUTP content). These data indicate that CHLA-01-MED and CHLA-01R-MED have intact sensing and response of p53 and p21 to an etoposide signal, although they were only mildly responsive to it in cytotoxicity assays in Figure 6, suggesting a different mechanism for the drug resistance of this medulloblastoma.

## Discussion

Medulloblastomas and other childhood cancers have been described in kindred with Fanconi anemia in the context of oncogenic *BRCA2* germline mutations (15–18). Our patient did not have Fanconi anemia as assessed by lack of phenotypic stigmata, normal DEB chromosome breakage testing, and usual tolerance to craniospinal irradiation and intensive chemotherapy. The patient’s family history was also not suggestive of Fanconi anemia. Additionally, this child was different from children with Fanconi anemia and childhood cancer in that he was 8 years old at medulloblastoma presentation, whereas medulloblastomas in *BRCA2*-mutated Fanconi anemia patients typically present before age 5 years (18). Indeed, our patient’s germline *BRCA2* 6174delT was only on one allele, with the other *BRCA2* allele remaining wildtype, whereas FANCD1 Fanconi anemia patients have biallelic deleterious germline mutations in *BRCA2*. Additionally, the anti-BRCA2-reactive band on western blotting of the patient’s brain tumor-derived cells suggests that the brain had at least one intact *BRCA2* gene. To our knowledge, this is the first reported child with medulloblastoma and a heterozygous germline *BRCA2* mutation and the first medulloblastoma described in the context of a *BRCA2* deleterious mutation that is not in a patient with Fanconi anemia.

The *BRCA2* 6174delT mutation causes frameshift and premature truncation of the BRCA2 protein. It is a deleterious mutation that in its heterozygous germline state is associated with high incidence of breast and ovarian cancers (*Am. J. Hum. Genetics* 62:676–689, 1998) (10). This, together with involvement in medulloblastoma of genes that function in DNA stability and repair (44–48) and the critical role *BRCA2* plays in neurogenesis and brain development (49, 50), renders it all the more interesting that this is the first report of a medulloblastoma associated with a heterozygous (non-Fanconi anemia) deleterious *BRCA2* germline mutation. It is also interesting that one of the two other medulloblastoma cell lines tested (UW-228-2) had an only minimal band reactive with the BRCA2 antibody on western blotting (Figure S1 in Supplementary Material), suggesting that BRCA2 needs to be examined in more detail in additional samples of medulloblastoma, especially when associated with inactivation of TP53 (49, 51). In view of the high incidence of *BRCA2* 6174delT carrier state in some populations (e.g., Ashkenazi Jews, ~0.9%), it will also be important to examine if children from families carrying the heterozygous *BRCA2* 6174delT mutation have higher incidence of medulloblastoma than non-carriers.

The finding of chromosome 17q gain in passage 2 cultured cells from the original tumor, cells obtained directly from the malignant pleural effusion at recurrence, and the cell line grown from this pleural effusion, but not in the direct FFPE tumor sections from the original resection may be due to differences in the method of detection. Its presence in malignant cells directly obtained from the pleural effusion at recurrence makes it unlikely that this mutation was acquired during growth in culture. Most likely is that cells harboring the 17q gain were a subclone in the original tumor and thus, not detected in the FFPE sections from the biopsy material. Since 17q gain was detected already in passage 2 cells grown from the original tumor (CHLA-01-MED), this subclone likely had a survival advantage in culture conditions, allowing its growth into the cell line. Presence of 17q gain also in the pleural effusion recurrence cells prior to culture suggest that this subclone was resistant to the chemotherapy and irradiation and may have contributed to the recurrence. Absence of the deletion in chromosome 11q in the FFPE tissue material is consistent with its absence in the CHLA-01-MED cell line from diagnosis. Its presence in the recurrence cells as well as in their cell line CHLA-01R-MED suggest that it became prominent during the evolution of the recurrence.

Isochromosome 17 is commonly seen in Group 3 and Group 4 medulloblastomas (6, 34–36). CGH analysis identified chromosome 17 aberrations with duplication of 17q in both original and recurrence cell lines. In the recurrence CHLA-01R-MED cell line, there were also an additional distinct breakpoint and a unique deletion at 17p13.2-ptel that includes the *TP53* locus. However, we did not detect any mutations in *TP53* nor were there any differences in p53 responses in our functional assays. Thus, the significance of these genomic lesions remains to be elucidated. High-level amplification of *MYC* was found in both cell lines. *MYC* elevation is more common in Group 3 medulloblastomas and is seen in up to 16.7% of them, but can also occur in some Group 4 or SHH tumors (52). Profiling of our patient’s medulloblastoma was consistent with Group 4. When *MYC* amplification occurs in a tumor with large cell/anaplastic histology and metastatic disease at diagnosis, the patients fare poorly, as did our patient (7, 53–55). Our combination of aCGH and RNASeq identified *CASC8* as a co-amplified but differentially expressed gene in the CHLA-01R-MED line. While the role of this transcript is unclear in medulloblastoma, it is part of a cluster of related lncRNAs that map to the *MYC* locus and are implicated in cancer risk (56).

According to prior classifications, the myogenic differentiation observed in this medulloblastoma would have classified it as a medullomyoblastoma (57). Many such tumors were described to have isochromosome 17 and amplification of *MYC*, similar to our findings in this case (57–59). Following the adoption of the 2007 WHO classification, such tumors are now called medulloblastomas with myogenic differentiation (60).

The Xp11.2 deletion targeting SSX6 (synovial sarcoma, X breakpoint 6; Gene ID: 280657) was found in the FFPE slides from the original tumor as well as the recurrence cells and the two cell lines. SSX genes were originally identified in synovial sarcomas with t(X;18) translocations, where SSX1 and SSX2 (and rarely SSX4) participate in oncogenic SYT-SSX fusions (61, 62). This medulloblastoma is the first report of a deletion involving SSX6 in any cancer. Since known oncogenic fusions of SSX genes function as aberrant transcriptional regulators, and the deletion at the SSX6 locus was found in both the original tumor and the recurrence, it will be informative to test if this lesion may have had a role in this child’s medulloblastoma. However, the RNASeq analysis did not detect any fusions that involved SSX6 or genes adjacent to the Xp11.2 deletion. Similarly, there was no evidence from either the karyotype or aCGH data for presence of known SSX fusion partners in either of the cell lines.

The del(11)(q13.3) that represented an 11q breakpoint targeting *FCHSD2* (FCH and double SH3 domains protein 2) was only found in the recurrent tumor cells, but not in the original tumor or its cell line. To date, this gene has not been described in the context of cancer, and its significance here is not known.

The combined RNA and aCGH analysis allowed identification of genes, which mapped to unique CHLA-01R-MED deletions and that were differentially expressed between the two cell lines (Table S1 in Supplementary Material). Genes in two regions were identified, 17p and 11q. The seven most significantly downregulated genes that mapped to the large chromosome 11q13.4-qtel deletion included *FAT3*. *FAT3* is a putative tumor suppressor that is a member of the cadherin family and is thought to function in interactions between neurites derived from specific subsets of neurons during development (63, 64). The 17p13.3-ptel deletion correlated with downregulation of ATPase, Na+/K+ Transporting Beta 2 Polypeptide (*ATP1B2*). The latter mediates cell adhesion of neurons and astrocytes, promotes neurite outgrowth, and in glioblastoma multiforme is an inhibitor of invasiveness (65, 66). Interestingly, GeneGO analysis of all genes upregulated in the recurrent line CHLA-01R-MED compared with the diagnosis line CHLA-01-MED identified the *WNT* signaling pathway as the most significant pathway (Figure S2 in Supplementary Material; Table S1 in Supplementary Material). The significance of the prominence of this pathway in the recurrence of this Group 4 medulloblastoma compared to the diagnosis tumor is not known.

Finally, another unique contribution of this work is the establishment and characterization of a pair of low-passage pediatric medulloblastoma cell lines from the original tumor (*MYC* amplified Group 4 medulloblastoma) and from its recurrence in the same patient, in which the germline *BRCA2* 6174delT mutation may have predisposed to the medulloblastoma. This pair of tumor cell lines may be of use to researchers working to enhance the understanding of medulloblastoma biology.

## Conflict of Interest Statement

The authors declare that the research was conducted in the absence of any commercial or financial relationships that could be construed as a potential conflict of interest.

## Supplementary Material

The Supplementary Material for this article can be found online at http://journal.frontiersin.org/article/10.3389/fonc.2015.00191

Figure S1 **CHLA-01-MED and CHLA-01R-MED express BRCA2 protein, although at lower amounts than some other brain tumor cell lines**. Western blot of whole cell lysates of the indicated cell lines was resolved by SDS-PAGE and hybridized with anti-BRCA2 antibody. GAPDH served as loading control. D283MED and UW-228-2 are medulloblastoma cell lines, CHLA-02-ATRT is an ATRT cell line (20), and LN229 is a GBM cell line.

Figure S2 **GeneGo analysis based on RNAs upregulated in CHLA-01R-MED relative to CHLA-01-MED cells identified Wnt signaling as the most significant pathway**.

Table S1 **Genes and pathways differentially expressed between the diagnosis cell line CHLA-01-MED and the CHLA-01R-MED cell line from the pleural fluid recurrence**. Differentially expressed genes and pathways were determined by combined RNA and aCGH analysis of the two cell lines, CHLA-01-MED and CHLA-01R-MED.
