# Supplementary figures and images for: Disseminated Medulloblastoma in a Child with Germline BRCA2 6174delT Mutation and without Fanconi Anemia
Source: Front Oncol. 2015 Aug 27;5:191. doi: 10.3389/fonc.2015.00191 (PMC4550790; doi:10.3389/fonc.2015.00191)

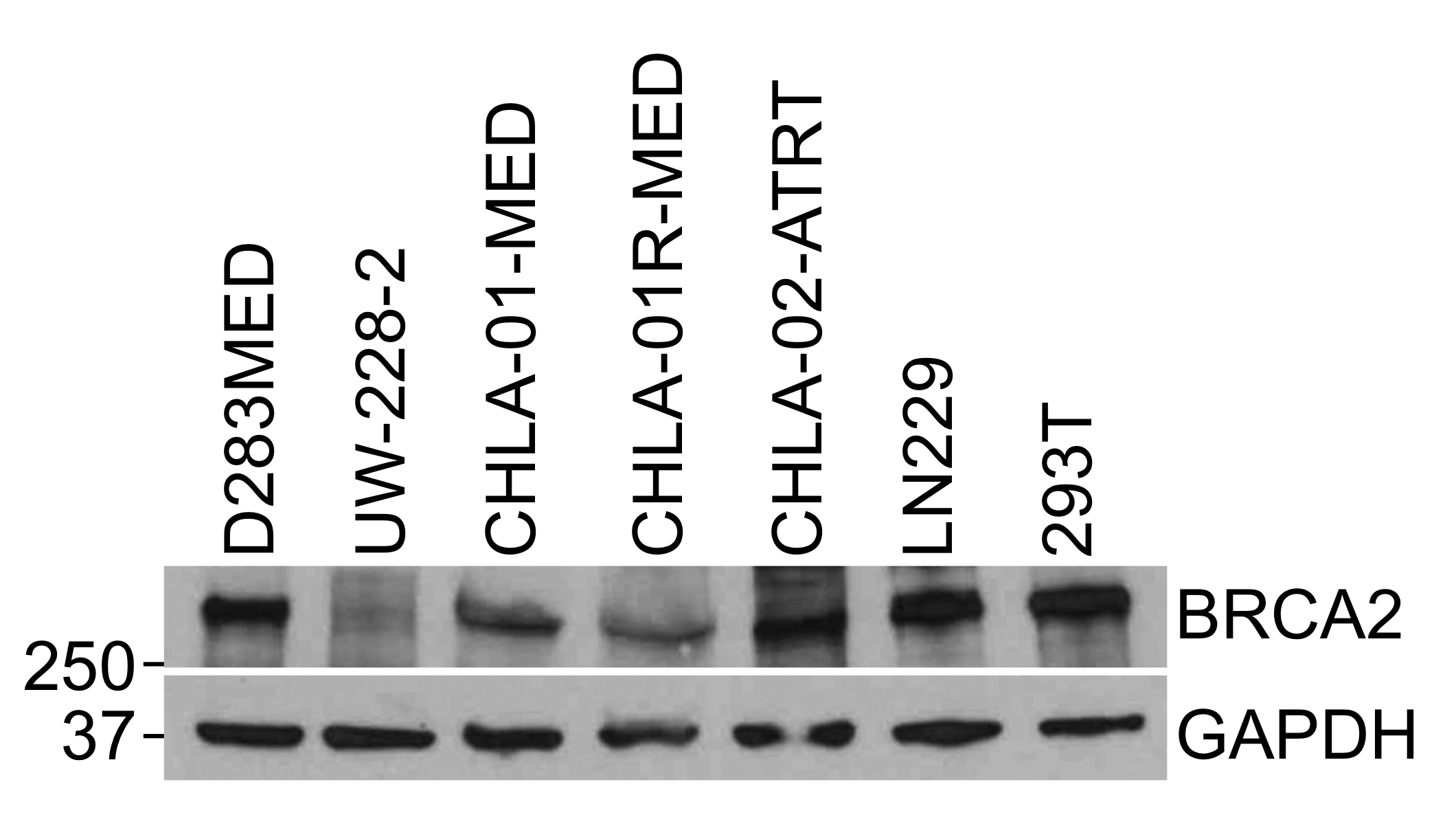

Supplement: Figure S1 — CHLA-01-MED and CHLA-01R-MED express BRCA2 protein, although at lower amounts than some other brain tumor cell lines. Western blot of whole cell lysates of the indicated cell lines was resolved by SDS-PAGE and hybridized with anti-BRCA2 antibody. GAPDH served as loading control. D283MED and UW-228-2 are medulloblastoma cell lines, CHLA-02-ATRT is an ATRT cell line (20), and LN229 is a GBM cell line. [file Image_1.JPEG]

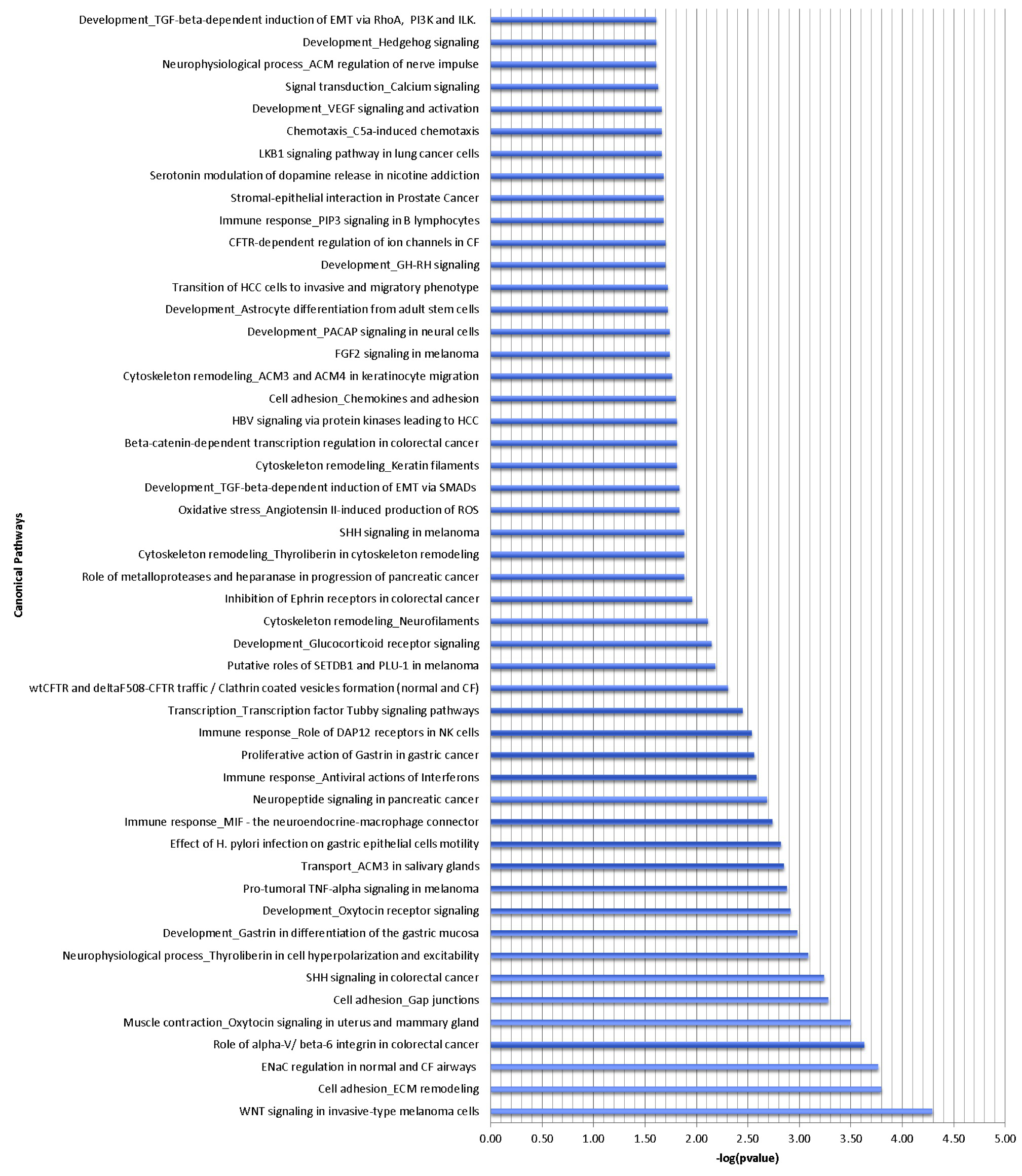

Supplement: Figure S2 — GeneGo analysis based on RNAs upregulated in CHLA-01R-MED relative to CHLA-01-MED cells identified Wnt signaling as the most significant pathway. [file Image_2.JPEG]
